# Improvement of a prediction model for heart failure survival through explainable artificial intelligence

**DOI:** 10.3389/fcvm.2023.1219586

**Published:** 2023-08-01

**Authors:** Pedro A. Moreno-Sánchez

**Affiliations:** Faculty of Medicine and Health Technology, Tampere University, Seinäjoki, Finland

**Keywords:** explainable artificial intelligence, medical XAI, heart failure, clinical prediction models, survival machine learning

## Abstract

Cardiovascular diseases and their associated disorder of heart failure (HF) are major causes of death globally, making it a priority for doctors to detect and predict their onset and medical consequences. Artificial Intelligence (AI) allows doctors to discover clinical indicators and enhance their diagnoses and treatments. Specifically, “eXplainable AI” (XAI) offers tools to improve the clinical prediction models that experience poor interpretability of their results. This work presents an explainability analysis and evaluation of two HF survival prediction models using a dataset that includes 299 patients who have experienced HF. The first model utilizes survival analysis, considering death events and time as target features, while the second model approaches the problem as a classification task to predict death. The model employs an optimization data workflow pipeline capable of selecting the best machine learning algorithm as well as the optimal collection of features. Moreover, different *post hoc* techniques have been used for the explainability analysis of the model. The main contribution of this paper is an explainability-driven approach to select the best HF survival prediction model balancing prediction performance and explainability. Therefore, the most balanced explainable prediction models are Survival Gradient Boosting model for the survival analysis and Random Forest for the classification approach with a c-index of 0.714 and balanced accuracy of 0.74 (std 0.03) respectively. The selection of features by the SCI-XAI in the two models is similar where “serum_creatinine”, “ejection_fraction”, and “sex” are selected in both approaches, with the addition of “diabetes” for the survival analysis model. Moreover, the application of *post hoc* XAI techniques also confirm common findings from both approaches by placing the “serum_creatinine” as the most relevant feature for the predicted outcome, followed by “ejection_fraction”. The explainable prediction models for HF survival presented in this paper would improve the further adoption of clinical prediction models by providing doctors with insights to better understand the reasoning behind usually “black-box” AI clinical solutions and make more reasonable and data-driven decisions.

## 1. Introduction

Cardiovascular diseases (CVD) are the global leading cause of death and disability with 17 million dead people approximately per year (31% of the total deaths globally). In this decade (2020–2030), an increase from 31.5% to 32.5% will result in 3.7 million additional deaths worldwide (1). In the US, the direct and indirect medical costs are expected to triple by 2030 respectively from $273 billion to $818 billion, and $172 billion to $276 billion. Therefore, it is crucial to develop preventive strategies to reduce CVD progression as well as minimizing the associated costs.

The term CVD involves different disorders of the heart and circulatory system manifested in different pathologies such as stroke, heart failure, or coronary heart disease. Heart Failure (HF) contributes significantly to CVD morbidity and mortality, as well as a large portion of related healthcare expenses (2). HF occurs when the heart is unable to pump blood effectively to the rest of the body and is accompanied by symptoms like shortness of breath or weakness (3). HF is often a consequence of other chronic diseases like diabetes or hypertension, as well as other patient conditions such as obesity, drug abuse or smoking (2). Globally, at least 26 million people are affected by HF, and presents a high mortality rate (about 50% of HF patients will die within 5 years) (4, 5). Given the vital importance of the heart for a person's life, the prediction of HF onset and its consequences (e.g., mortality) has become a priority for doctors and healthcare providers, not only due to its implications for patient health but also because of the increased resources required for patient follow-up (e.g., economic, humans resources, etc.). However, despite this urgent need, the clinical practice has so far failed to achieve high accuracy in these tasks (6).

As a result, modelling survival patients with HF remains currently challenging concerning the early identification of clinical factors associated with its mortality and achieving high classification accuracy (7). Currently, angiography is considered the most precise method for predicting CVD. However, its high cost poses a barrier to access, particularly for low-income families (8). In this context, the increasing availability of electronic data implies an opportunity to democratize access to prediction models for HF survival. Machine Learning (ML) and Artificial Intelligence (AI) have emerged as promising tools in healthcare, supporting clinicians in detecting disease patterns, predicting risk situations for patients, and extracting clinical knowledge from vast amount of data. Computer-aided diagnosis systems, through ML algorithms implementation, offer a diagnosis of complex health issues with good accuracy and efficiency (9, 10). Therefore, ML is seen as a means to provide healthcare professionals with appropriate solutions to discover latent correlations between HF survival and clinical indicators enabling early detection of those patients at risk.

Nevertheless, when the decisions made by computer-aided diagnosis systems affect the patient's life, their use in the clinical routine is not straightforward. In the healthcare domain, clinicians require far more information from the prediction models than a simple binary decision. Therefore, providing explanations that support the outputs of ML models is crucial to ensure their adoption. The field of eXplainable Artificial Intelligence (XAI) has emerged to address this requirement. XAI is defined as follows: “Given an audience, an explainable Artificial Intelligence is one that produces details or reasons to make its functioning clear or easy to understand” (11). In the medical context, the lack of explainability in certain prediction models needs be addressed, as clinicians find it challenging to trust complex ML methods that require high technical knowledge (12). Thus, XAI enables healthcare experts to make more informed and data-driven decisions, providing personalized and trustworthy treatments and diagnoses (13). However, XAI is not a “one-size-fits-all” solution because an inherent tension between accuracy and explainability appears depending on ML models employed. Typically, the best-performing models are more complex and less interpretable (e.g., ensemble trees or neural networks) and vice versa. Additionally, despite the benefits XAI might bring to ease the path for clinical adoption, ML models, especially in the healthcare domain, can often be riddled with different issues related to ethics (fairness, non-discrimination, accessibility) and regulation (accountability, privacy, and data governance) that hinder their uptake by doctors and healthcare professionals (14).

This paper aims to describe the development of two prediction models for HF survival, achieving a balance between prediction performance and explainability. The first model utilizes survival analysis, considering death events and time as target features, while the second model approaches the problem as a classification task to predict death. In addition, the paper analyzes the influence of the different clinical indicators on the prediction results by applying explainability *post hoc* techniques to the model. To develop the explainable prediction model, an optimization data pipeline is used to select different model parameters such as the ML algorithm for the survival analysis or classification problem, and the selected features that indicate the best classification performance. The study follows the standardized practices of reporting prediction models in medicine by adopting the TRIPOD statement guideline, which includes a 22-item checklist (15) provided as Supplementary Material.

The remainder of this paper is structured as follows: Section 2 provides a review of related works that have developed an HF survival prediction model with the same dataset used in this paper. Section 3 describes the dataset, the different ML algorithms, feature selection methods, metrics employed in this work, along with the optimization pipeline employed to build the predictive model. Section 4 presents the evaluation results in terms of prediction performance of both approaches (survival analysis or classification problem) and explainability, and the analysis of the importance of features. In section 5, the obtained results are discussed. Finally, Section 6 includes the conclusions drawn from the work.

## 2. Related works

The demand for tools that increase the accessibility of AI to healthcare professionals is steadily growing, as AI solutions usually require expert knowledge of ML algorithms (16). This need is particularly crucial in precision medicine, where disease diagnosis requires interpretable and transparent information (17). XAI solutions, aimed at providing healthcare professionals with prediction models' global explanations, have been used for over a decade. Transparent models such as logistic and linear regression, naïve Bayes, decision tree, or k-nearest neighbors have been employed in various clinical fields, including urology (18, 19), cardiology (20), toxicology (18, 21), endocrinology (22), neurology (23), psychiatry (24, 25), occupational diseases (26), knee osteoarthritis (27), breast cancer (28), prostate cancer (29), severity of Alzheimer's disease (30), diabetes (31) and mortality rates of CVDs such as myocardial infarction or perinatal stroke (32, 33). Model-agnostic explainability solutions such as SHAP (SHapley Additive exPlanations) or MUSE (Model Understanding through Subspace Explanations) have been applied to complex AI solutions based on deep learning to diagnose depression (34), predict chronic kidney disease (35), or detect acute intracranial hemorrhage in images (36).

HF outcome prediction is critical to accurately apply available therapeutic options, ranging from pharmacologic to highly invasive mechanical ventricular assistance and cardiac transplantation (37). ML techniques can be valuable in early-stage risk prediction using the variables derived from the complex and diverse EHR data of patients. Several accurate methods, such as the ADHERE model (38) and the Seattle Heart Failure Model (39), have been developed in the last decade to estimate the risk of death for patients with HF. However, these models were unintuitive and relied on extensive medical records, making them challenging to apply in a clinical setting (40). Other studies have been developed to classify CVD diseases and to accurately predict abnormalities in the heart or its functioning (41–43). Various ML algorithms have been employed in CVD prediction models, including Support Vector Machines, Logistic Regression, Artificial Neural Networks, Random Forest, Decision Tree, Ensemble Learning approaches, Deep Neural Networks, Fuzzy experts system, or K-nearest Networks (44). However, modelling survival heart failure is still lacking in terms of driving factors identification, since existing models present limited interpretability of their prediction variables (45, 46). Another issue observed in the literature is the lack of consensus regarding the relevance of HF indicators, as studies employ different datasets that affect the models' reliability to be deployed in clinical routine (37, 47). Consequently, partial approaches tackle the model's effectiveness through cohorts with specific types of patients (e.g., elderly o diabetic) (48, 49), although their models developed have not achieved optimal performance (50, 51).

Therefore, to ensure an objective comparison with other HF prediction models, it is essential to maintain homogeneity regarding the dataset. The dataset released by Ahmad et al. (52) in the UCI public repository (53) allows for benchmarking the other authors' prediction models. Table 1 shows the most recent studies that employ Ahmad's dataset to build a prediction model for HF survival. However, the different works reviewed reflect two approaches in the built of the survival prediction model, i.e., through a classical classification machine learning problem where the target feature is the event of death, or through a survival analysis where the relation between the event of death and the censored time is analyzed. The reviewed works that tackled the prediction as a classification problem, which is the major option among the reviewed works, are sorted in a descendant order of accuracy (Acc.), and additional information for each study is expressed such as Sensitivity (Sens.), Specificity (Spec.), f1-score (F1), and Precision (Prec.); the number of features (#Feat.) after applying feature selection; and the Machine Learning (ML) technique. The comparison table also indicates if the studies consider the feature “time” in their modeling since we have detected that some studies leave “time” out of their datasets.

**Table 1:** Classification results of related works and ML classifiers (best ones in italic).

Author	Acc. (Bal Acc)	Sens.	Spec.	Prec.	F1	#Feat	ML classifiers
Kumar et al. (54)	0.96	0.93	–	0.95	0.94	5^b^	*Random Forest*, XGBoost, Decision Tree, Logistic Regression, Support Vector Machine, K-Nearest Neighbour, Gradient Boosting, Stochastic Gradient Descent, Gaussian Naïve Bayes
Kaddour (7)^a^	0.90 (0.91)	0.93	0.90	–	–	4	FeedForward Neural Network, *Deep Neural Network*
Ishaq et al. (44)^a^	0.88	0.89	–	0.89	0.89	12^b^	*Random Forest*, XGBoost, Decision Tree, AdaBoost, Extra Trees, Logistic Regression, Support Vector Machine, Gradient Boosting, Stochastic Gradient Descent, Gaussian Naïve Bayes
Sandhu et al. (55)	0.88	0.83	–	0.81	0.84	12^b^	*Bayesian generalized linear model*, Artificial Neural Network, Bagged CART, Support Vector Machine, Random Forest, Decision Tree
Kucukakcali et al. (56)	0.87 (0.82)	0.69	0.95	–	0.77	12^b^	*Associative Classification*
Rahayu et al. (57)	0.83	–	–	–	–	12^b^	Random Forest, Decision Tree, K-Nearest Neighbour, *Support Vector Machine*, Artificial Neural Network, Naïve Bayes
Srujana et al. (58)	0.85	–	–	–	–	3^b^	*Random Forest.*
Özbay et al. (59)	0.84	–	–	–	–	–^b^	Logistic Regression, Naïve Bayes, Support Vector Machine, K-Nearest Neighbour, *Bagged Trees*, Boosted Trees, Multilayer NN
Chicco and Jurman (60)	0.84	0.78	0.86		0.72	3^b^	*Random Forest*, Gradient Boosting, Support Vector Machine with radial kernel
Gürfidan and Ersoy (61)	0.83	–	–	–	–	12^b^	*Support Vector Machines*, Logistic Regression, Decision Tree, K-Nearest Neighbour, Linear Discriminant Analysis, Gaussian Naïve Bayes
Muntasir et al. (62)	0.83	0.86	–	0.90	0.88	12^b^	Decision Tree, Logistic Regression, Gaussian Naïve Bayes, *Random Forest*, K-Nearest Neighbour, Support Vector Machine
Wilstup and Cave (40)	0.82	–	–	–	–	3^b^	*Cox models plus symbolic regression*
Khan et al. (63)	0.81	0.82	0.74	–	–	5	Support Vector Machine (Kernel Linear, Radial Basis Function, *Cubic* and Quadratic)
Taj et al. (64)	0.72	–	–	–	–	7^b^	*Fuzzy Preti nets plus Rough Set Theory*

Acc, accuracy; Bal Acc, Balanced Accuracy; Sens, Sensitivity; Spec, Specificity; F1, f1-score; Prec, Precision; the number of features for modelling excluding target (#Feat.).

^a^ Studies that perform the best classifier over unseen new data.

^b^ Time is considered as a feature.

It should be noted that since the dataset is imbalanced in its target feature, some works consider also balanced accuracy in their metrics. In addition, there are other works that address the imbalance by applying auxiliary techniques such as SMOTE (65, 66) to equalize the number of instances in the target feature. However, as we have not applied such techniques, we have not included the results of these works in the table to avoid any misunderstanding in the comparison. Additionally, the application of these techniques, while improving the performance of the model in controlled environment, does not reflect the true population where the distribution of the classes in the target feature is unequal, implying a risk of overfitting and bias that lead to misclassification when the model is applied in a real-world setting.

## 3. Material and methods

### 3.1. Heart failure survival dataset

The dataset employed in this paper, released by Ahmad et al. (52), consist of the medical records of 299 patients (194 men and 105 women) who suffered an HF episode. The dataset was collected from April to December 2015 at the Faisalabad Institute of Cardiology and at the University Allied Hospital in Faisalabad (Punjab, Pakistan). Pakistan is among the countries where prevalence of CHD is increasing significantly reaching about 200,000 per year, i.e., 410/100,000 of the population. Additionally, this region is characterized by lack of exercise, poor health care policies, and poor and oily diet which are different from other of South Asia like India, Bangladesh, Nepal and Sri Lank. Faisalabad, specifically, is the country's third most populous city making obtained the result potentially representative of the urban population of Pakistan.

The dataset comprises 7 numerical and 5 categorical or nominal features along with one binary target feature (“death event”). This dataset presents an imbalance concerning its target feature since 203 out of the 299 instances belong to patients who survived HF (“death event” = 0), and the remaining 96 instances represent deceased patients (“death event” = 1). All instances of the dataset are entirely complete with no missing values in any of their features. The dataset description is presented in Table 2.

**Table 2:** Dataset's features description.

Id	Feature (units)	Range (mean ± std)/binary values (number of instances per class)
1	Age (years)	40–95 (60.83 ± 11.89)
2	Anaemia (boolean)	0 (170) or 1 (129)
3	High Blood Pressure (boolean)	0 (194) or 1 (105)
4	Creatinine phosphokinase-CPK (mcg/L)	23–7,861 (581.83 ± 970.29)
5	Diabetes (boolean)	0 (174) or 1 (125)
6	Ejection fraction (percentage)	14–80 (38.08 ± 11.83)
7	Sex (boolean)	0 (194-Men) or 1 (105-Women)
8	Platelets (kiloplatelets/ml)	25,100–8,50,000 (2,63,358.03 ± 97,804.23)
9	Serum creatinine (mg/dl)	0.50–9.40 (1.39 ± 1.03)
10	Serum sodium (mEq/L)	113–148 (136.62 ± 4.41)
11	Smoking (boolean)	0 (203) or 1 (96)
12	Time-Follow up period (days)	4–285 (130.26 ± 77.61)
13	[Target] Death event (boolean)	0 (203) or 1 (96)

An exploratory data analysis over the numerical variables reveals that the features creatinine_phosphokinase, ejection_fraction, platelets, serum_creatinine, and serum_sodium presents outliers in their values distribution according to a greater distance than 1.5 IQR from the 3rd quartile (see Supplementary Figure S1). Due to the low number of instances in the dataset (299), removing the instances that contain outliers might be detrimental for the model performance. Therefore, we adopt the winsorization at percentiles strategy, which involves replacing the extreme values beyond a certain percentile with the nearest value within that percentile. In this case, we set an upper limit of 90, which only affects the feature creatinine_ phosphokinase due to its outlier distribution. Consequently, the statistical summary (range, mean ± std) of its values becomes 23–1,203 (416.77 ± 369.20).

Another aspect derived from the features in the dataset is the relation between the feature “time” and the target feature “death”. The target feature indicates whether the patient died during the follow-up period, while the feature “time” represents the number of days until the ocurrence of death or, in the case of surviving patients, the censored time according to the duration in days of the follow up. Consequently, if the prediction model is addressed as a classification machine learning model the feature “time” could be considered a surrogate variable for the target feature. Additionally, both features present a significant correlation (0.53) as Supplementary Figure S2 shows. Therefore, excluding the feature “time” from the model development in the classification problem might be recommended. However, most of the related works considered in our analysis did not account for this surrogate phenomenon and include “time” as a feature in their modeling. With the aim of comparing our proposed optimization pipeline which balances interpretability and prediction performance, we address both approaches found in the literature (survival analysis and classification problem). Subsequently, the optimal models obtained are analyzed trough an explainable perspective.

### 3.2. Ensemble tree algorithms

Ensemble trees techniques, by weighting and combining various models generated from a base decision tree, typically offer reasonably good accuracy in classification tasks and are commonly used in different research fields such as health, economy, biology, and more (67). These ensemble methods not only outperform the weak base classifier but also help mitigate challenges such as class imbalance or the curse of dimensionality (67). However, due to the lack of explainability capabilities, ensemble trees might be avoided by professionals who needs to interpret the predictions. Consequently, *post hoc* explainability techniques are needed to interpret the black-box behavior of ensemble trees. The different ensemble trees algorithms employed in this work are described as follows:

• *Random Forests*: Random Forest is one of the most widely used ensemble tree methods due to its good predictive performance and the capability to handle datasets of different sizes. To train its base classifier (decision tree), Random Forest employs the bagging method, which selects a random group of features at each splitting in its nodes (67).
• *Extreme Randomized Trees (Extra Trees)*: Extra Trees is another ensemble method that improves the accuracy of tree-based bagging classifiers by selecting random cut-points in the node splitting process and using the entire training dataset for all the base classifier trees. This method is similar to Random Forest, but it introduces additional randomness in the node splitting process, resulting in a more diverse set of trees. This additional randomness can help to reduce overfitting and improve generalization performance (68).
• *Adaptive Boosting (AdaBoost)*: AdaBoost is a boosting ensemble method that focuses on training the model on misclassified instances, which receive modified weights over successive iterations. The base classifiers also receive weights based on their performance, which influence the classification output of a new instance. This technique results in a strong classifier that combines the output of multiple weak classifiers (67).
• *Gradient Boosting*: Gradient boosting trains their base classifier over the residual errors from the precedent classifiers, hence, reducing the classification error. The overall classification result is obtained through a weighted average of all base classifiers' results (69).
• *eXtreme Gradient Boosting (XGBoost)*: XGBoost applies several optimizations and regularization processes to the gradient boosting algorithm in order to increase the speed and performance as well as make the algorithm simpler and more generative (66).

### 3.3. Machine learning algorithms for survival analysis

Currently, there is a growing number of ML algorithms for survival analysis that provides data scientists with alternatives to the regular survival techniques such as Kaplan-Meier curves and Cox proportional hazard. In this work we consider several ML techniques aimed for survival analysis that are available in the python library scikit-survival (70).

- *Cox proportional hazard (CPH)* is a semiparametric technique used to determine the influence of a specific set of covariates (also known as features) on the risk or hazard of an event, such as death in our context. It calculates the hazard for a patient based on a combination of the population's baseline hazard (which varies over time) and the patient's static predictor covariates, each multiplied by their respective coefficients. In this work, we also considered penalization mechanisms such as Elastic Net, which is recommended for addressing situation with high-multidimensionality and high correlation (71).
- *Random Survival Forest (RSF)* is an extension of the random forest method that can capture complex relationships between the predictors and survival without requiring prior specification. RSF can handle multiple features, noise features, as well as complex, nonlinear relationships between features without the need for prior specification. The algorithm builds survival trees by recursively partitioning the feature space using binary splits to form groups of subjects who are similar according to the survival outcome (72).
- Extra Survival *Trees* is an extension of the Extremely Randomized Trees that consider censoring and is used to model the relationship between the survival time and a set of features. It is a non-parametric method that recursively partitions the data into homogeneous subgroups based on the features. The resulting tree is used to predict the survival time of new observations (73).
- *Gradient Boosted Models (GBMs)* for survival analysis is an extension of the Gradient Boosted Trees models. GBMs are constructed sequentially in a greedy stagewise fashion, and the base learners are regression trees that try to minimize a loss function that depends on the problem (71).
- *Survival support vector machines (SSVMs)* are an extension of the standard SVM and aim to find a hyperplane that separates the data into two groups: those that have experienced the event of interest and those that have not. The hyperplane is found by maximizing the margin between the two groups (71).

### 3.4. Explainability techniques for ML

In terms of explainability, decision trees are considered as “transparent” models due to their graphical structure and decomposability, which provides a fully interpretable functionality, making them suitable for domains such as healthcare where understanding the outputs of ML models is necessary. Conversely, ensemble trees and other ML models such as SVM require the support of *post hoc* explainability techniques since their classification decision is based on the combination of multiple decision trees' results. *Post hoc* explainability techniques offer understandable information about how an already developed model produces its predictions by employing common methods that humans use to explain systems, such as visual, local, or feature relevance explanations (11). These kinds of techniques are also used in this research, and are described below.

- *Feature Permutation Importance: Permutation* feature importance measures the increase in the prediction error of the model after permuting a specific feature's values (74). This model-agnostic technique (non-dependent on the ML algorithm to explain) indicates a feature as important if the error increases by shuffling the feature's values a specific number of times. Vice versa, if the error does not change by shuffling the feature's values, the feature is “unimportant”.
- *Partial Dependence Plot: Partial Dependence Plot (PDP)* is a *post hoc* explainability technique that provides a visual explanation by showing the marginal effect of a given feature on the predicted outcome (75). The concept of marginal effect indicates how a dependent variable changes when a specific independent variable changes its values, while keeping other covariates constant. Therefore, PDP can be used as a model-agnostic method for global explainability to determine the average effect of a feature over a range of different observed values. For classification tasks, such as the one performed in this study, PDP displays the probability (average and confidence interval) for a certain class as a function of the feature value. PDP also offers a multivariate option, where, for instance, the marginal effect of two features can be analyzed over the output probability.
- *Shapley Values*: The Shapley Additive exPlanations (SHAP) technique is a model-agnostic method that combines explanations by example with feature relevance. The technique computes an additive feature importance score for each individual prediction with local accuracy and consistency (76). SHAP computes the contribution of each feature to the predicted outcome/class by applying coalitional game theory (77). In classification tasks, the SHAP technique computes a signed importance score that indicates the weight of a feature towards the predicted outcome as well as its direction, where positive values increase the probability of class 1 and negative ones decrease such probability. In addition, SHAP can also be applied for survival analysis by using the library SurvSHAP (78) which can discover patterns in the predicted survival curves that would identify significantly different survival behaviors, and utilizing a proxy model and SHAP method to explain these distinct survival behaviors.

### 3.5. Feature selection

Feature selection cannot be considered as a specific explainability technique; however, it can enhance model explainability since when performed during the data preprocessing phase, those unimportant features that bring non-relevant information to the classification are removed. Feature selection also allows decreasing overfitting in models' prediction and reducing computing time. Moreover, searching for a relevant features subset involves finding those features that are highly correlated with the target feature, but uncorrelated with each other (79).

Generally, there are three types of feature selection methods: filters methods where intrinsic properties of data justify the inclusion of an attribute or a subset of attributes; wrappers methods, which are similar to filters but utilize a classification algorithm; and embedded methods that combine filter and wrapper to achieve a better classification performance. Concerning filter methods, different techniques are applied depending on the data type of the features and the target variable (80). For instance, ANOVA correlation coefficients are used in the case of numerical input and categorical output, and Chi-Squared test when both categorical input and output occur. Mutual information is another filter method applied when the output variable is categorical but does not depend on the input data type. As regards wrapper methods, one of the most frequently employed is Recursive Feature Elimination (RFE) that use an estimator, like logistic regression, to reduce recursively the features in a dataset by discarding those features with the smallest weights during recursive iterations. These four methods have been considered in this research.

### 3.6. Performance metrics

This paper considers different metrics to evaluate the performance of the prediction model when tackling the prediction of the death event as a classification problem or as a survival analysis, as well as its explainability. Table 3 summarize the formulas of some these metrics.

**Table 3:** Classification and explainability metrics formulas.

Ensemble trees algorithm	Description
Accuracy	$\frac{\left(TP+TN\right)}{\left(TP+TN+FP+FN\right)}\;(1)$
Sensitivity/Recall	$\frac{TP}{\left(TP+FN\right)}\;(2)$
Specificity	$\frac{TN}{\left(TN+FP\right)}\;(3)$
Balanced accuracy	$\frac{Sensivity+Specificity}{2}\;(4)$
Precision	$\frac{TP}{\left(TP+FP\right)}\;(5)$
F1-Score	$2*\frac{Precision\;*\;Sensivity}{Precision+Sensivity}\;(6)$
Interpretability (I)	$\frac{masked\;features}{total\;input\;features}\;(7)$
Interpretability-accuracy index (IAI)	I∗Acc(9)
Interpretability-concordance index (ICI)	I∗Cindex(9)

(TN as true negative, FN as false negative, FP as false positive, and TP as true positive)

Accuracy measures the rate of true predictions in all classifications made with a dataset and it is a recommended metric for dealing with balanced datasets. However, the dataset used in this work is not balanced in its target feature, thus, the accuracy metric can give a wrong idea about the model's classification performance. Thus, the balanced accuracy gives a better insight since it accounts for the imbalance in classes. The rest of the metrics considered are especially useful when evaluating a classification model within the healthcare domain where false positive and false negative are important (18).

For survival analysis problems, the most commonly used metrics is the concordance index (C-index), which measures the rank correlation between predicted risk scores and observed time points. It calculates the ratio of correctly ordered (concordant) pairs to comparable pairs. The C-index ranges from 0 to 1, where a value of 1 indicates perfect concordance between risks and event times, a value of 0 indicates perfect anti-concordance between risks and event times, and a value of 0.5 indicates random assignment (81). However, C-index can be overly optimistic with increasing censoring, and it may not be useful when a specific time range is of primary interest (e.g., predicting death within 2 years). o address these limitations, the C-index based on inverse probability of censoring weights (C-index IPCW) is employed. The C-index IPCW is unbiased and consistent, as it does not depend on the distribution of censoring times and provides a population concordance measure free of censoring. In addition, when extending the receiver operating characteristics (ROC) curve to continuous outcomes, such as survival time, a patient's disease status is typically not fixed and changes over time, thus, the sensitivity and specificity become time-dependent measures. In this work, we consider cumulative cases (individuals who experienced an event prior to or at time *t*) and dynamic controls (those without event at time *t* yet) at a given time point. o address these limitations, the C-index based on inverse probability of censoring weights (C-index IPCW) is employed. The C-index IPCW is unbiased and consistent, as it does not depend on the distribution of censoring times and provides a population concordance measure free of censoring.

Additionally, as one of the goal of this work is to identify the most balance prediction model in terms of explainability and prediction performance, specific metrics for explainability are needed, such as Interpretability which is proposed by Tagaris et al. (82). The interpretability of the model, I (model), is defined as the percentage of masked features that do not bring information to the final prediction result, divided by the total number of features of the dataset. The Interpretability-Accuracy Index is a metric used to identify the model that achieves the best balance between accuracy and interpretability. It serves as a measure to assess the trade-off between the two factors and determine the optimal model. In addition, to quantify the balance between interpretability and survival prediction, we introduce the Interpretability-Concordance index. This index evaluates how well a model performs in terms of predicting survival outcomes while taking into account its level of interpretability. It provides a measure of the model's ability to strike a balance between accurate predictions and the ability to explain its reasoning. These indices are valuable tools in evaluating and selecting models that not only deliver accurate predictions but also provide interpretable insights, thus aiding in decision-making processes in various domains.

### 3.7. Data workflow optimization pipeline

**Figure 1:**
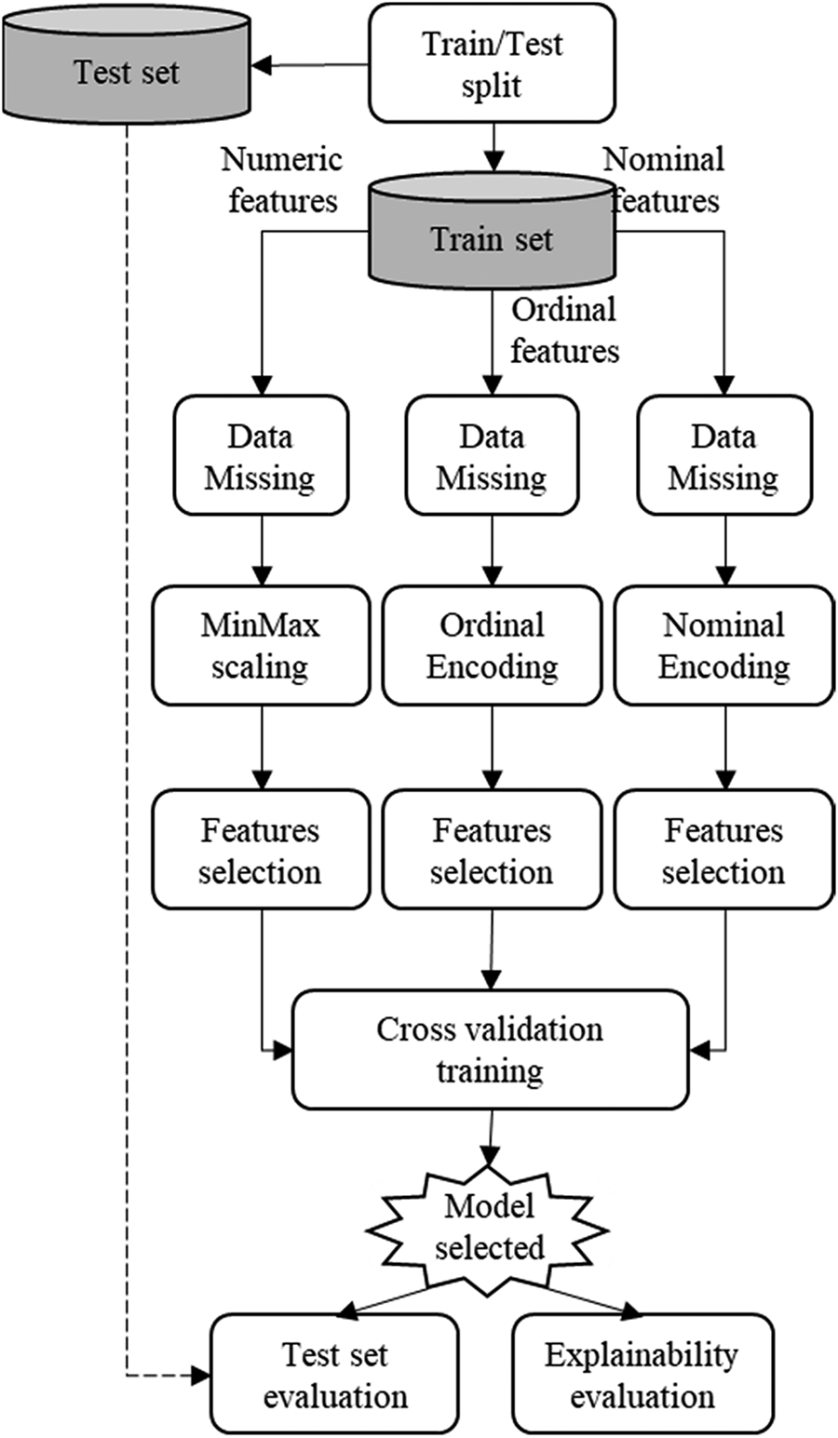
SCI-XAI data workflow optimization pipeline.

To develop the explainable prediction model for HF survival, we used the automated data workflow pipeline named SCI-XAI published in (83).^1^ As shown in Figure 1, the SCI-XAI pipeline utilizes the GridSearchCV module of python scikit-learn package (84) which applies a brute force algorithm to find the optimal combination of classification ensemble tree technique or survival machine learning techniques, the number of features selected, and of feature selection method in terms of classification performance.

As a first step, the original dataset is divided into two sub-datasets: a training set with 280 instances and a test set with and 120 instances. A split ratio of 70/30 is adopted with a stratification approach that ensures the same proportion of the target feature (“Death_event”) in both sets. This initial split is intended for building the prediction model exclusively using the train set's instances. Subsequently, the model's performance is evaluated using unseen new data stored in the test set. This approach prevents any influence of the test set instances o feature selection and classifier training modules. The data preprocessing phase encompasses modules such as data missing imputation, normalization (in case of numerical features), encoding (for nominal and ordinal features), and finally the feature selection. This data preprocessing module handles features depending on their type (numerical, nominal and ordinal). The modelling or training phase is carried out using a 5-fold cross-validation approach to fit different types of classification ensemble tree technique or survival machine learning techniques on the training data to identify the best model, which is subsequently evaluated in terms of classification and explainability. It is important to note that data imputation module is not applied in this work, since the dataset used does not contain any missing data.

## 4. Results

### 4.1. Classification performance

The results obtained from applying different ensemble tree learning algorithms in the 5-fold cross-validation training module are presented in Table 4 along with the number of nominal and categorical features selected. The Table 4 displays the best performance of each classifier that intrinsically selects a group of features by using the SCI-XAI framework. The classifier with best classification performance in the cross-validation in terms of balanced accuracy is Random Forest (mean: 0.74, std: 0.03), followed by XGBoost (mean: 0.73, std: 0.07) and Extra Tree (mean: 0.72, std: 0.03). Finally, the optimal classifier is applied to new unseen data, and the results are also shown in Table 4. The Random Forest classifier achieves the highest classification results, with a balanced accuracy value of 0.71.

**Table 4:** Classification results of the training set (cross-validation approach) and test set with new unseen data.

Classifier	Training set (cross-validation approach)							Test set (new unseen data)					
	Acc.	BAcc	Sens.	Spec.	Prec.	F1	#F	Acc.	BAcc	Sens.	Spec.	Prec.	F1
Random Forests	0.78 (0.02)	0.74 (0.03)	0.64 (0.09)	0.84 (0.05)	0.66 (0.06)	0.65 (0.05)	2(N), 1(C)	0.75	0.71	0.58	0.83	0.62	0.60
Extra Trees	0.76 (0.04)	0.72 (0.03)	0.61 (0.07)	0.83 (0.08)	0.66 (0.10)	0.63 (0.04)	3(N), 1(C)	0.74	0.70	0.59	0.81	0.61	0.59
AdaBoost	0.73 (0.06)	0.69 (0.05)	0.50 (0.06)	0.88 (0.05)	0.68 (0.09)	0.57 (0.06)	3(N), 1(C)	0.73	0.64	0.48	0.85	0.60	0.54
Gradient Boosting	0.76 (0.04)	0.71 (0.05)	0.59 (0.11)	0.84 (0.04)	0.63 (0.04)	0.60 (0.07)	3(N), 1(C)	0.72	0.64	0.41	0.87	0.60	0.49
XGBoost	0.77 (0.04)	0.73 (0.05)	0.65 (0.11)	0.82 (0.07)	0.65 (0.09)	0.64 (0.06)	3(N), 2(C)	0.74	0.69	0.55	0.83	0.61	0.58

Acc, accuracy; Bacc, balanced accuracy; Sens, sensitivity; Spec, specificity; Prec, precision; F1, F1-score; #F, number of features; N, numerical; C, categorical. The results indicates mean and standard deviation in parenthesis.

### 4.2. Feature selection

The SCI-XAI pipeline not only identifies the best performance for each classifier but also determines the optimal number of features that contribute to that performance. Acc: Accuracy, Bacc: Balanced accuracy, Sens: Sensitivity, Spec: Specificity, Prec: Precision, F1: F1-Score, #F: Number of features. (N): Numerical, (C): Categorical. The results indicates mean and standard deviation in parenthesis.

Table 5 shows the number of selected numerical and categorical features, along with their names and the techniques employed for feature selection, i.e., ANOVA, chi-squared, Mutual information (mut-inf), or recursive feature elimination (RFE). Among the classifiers, the lowest number of features is obtained with Random Forest where two numerical features are selected by ANOVA namely “ejection_fraction”, and “serum_creatinine” and only one categorical feature “sex” is selected by the mutual information method.

**Table 5:** Numerical and nominal features selected (# feats: number of features).

Classifier	# Feats	Numerical features (select method)	# Feats	Categorical features (select method)
Random Forests	2	“ejection_fraction”, “serum_creatinine” (ANOVA)	1	“sex” (mut-inf)
Extra Trees	3	“ejection_fraction”, “serum_creatinine”, “serum_sodium” (ANOVA)	1	“sex” (mut-inf)
AdaBoost	3	“ejection_fraction”, “serum_creatinine”, “serum_sodium” (mut-inf)	1	“anaemia” (chi-squared)
Gradient Boosting	3	“ejection_fraction”, “serum_creatinine”, “serum_sodium” (ANOVA)	1	“sex” (mut-inf)
XGBoost	3	“ejection_fraction”, “serum_creatinine”, “serum_sodium” (ANOVA)	2	“sex”, “anaemia” (mut-inf)

### 4.3. Explainability performance

When determining the best combination of relevant features for each classifier, it is also possible to evaluate the explainability. The results of these metrics can be found in Table 6, which shows that all of the techniques considered in the SCI-XAI pipeline reduce the number of training features by more than 50%. Considering IAI as the metric that gives a balanced measure between interpretability and accuracy, prediction model built by Random Forest with an IAI value of 0.56 can be denoted as the most balanced model among those evaluated in terms of explainability and classification accuracy. Therefore, the prediction model built with Random Forest and its group of selected features (“serum_creatinine”, “ejection_fraction”, and “sex”) is used for conducting the explainability analysis when tackling the survival prediction as a classification problem.

**Table 6:** Explainability metrics results.

Classifier	Interpretability	IAI
Random Forests	0.73	0.56
Extra Trees	0.64	0.48
AdaBoost	0.64	0.47
Gradient Boosting	0.64	0.48
XGBoost	0.55	0.42

### 4.4. Survival prediction model performance

The SCI-XAI methodology is applied to the previously described algorithms, namely Cox-proportional hazard (with and without the Elastic Net approach), Random Survival Forest, Extra Survival Trees, Survival support vector machines, and gradient boosted models for survival analysis. Table 7 illustrates the optimal performance of each survival ML technique based on the various considered metrics. The pipeline utilizes the c-index to identify the most effective combination of selected features for optimal survival prediction. This combination is then evaluated using the test set through metrics such as c-index, c-index IPCW, and AUCD_ROC. The Interpretability Concordance Index is also calculated to determine the model that strikes the best balance between interpretability and prediction performance. Based on the results, the Gradient Boosting models emerge as the best algorithm, both when trained and tested. The SCI-XAI output indicates that the optimal combination for the GBM model comprises two numerical features and five nominal features, resulting in a c-index of 0.714 (0.013) for the training set and values of 0.724, 0.762, and 0.748 for c-index, c-index IPCW, and AUCD-ROC respectively in the test set. However, this model exhibits a low interpretability concordance index of 0.298. Consequently, a manual inspection of all combinations for the GBM model is conducted to identify a more balanced model in terms of interpretability and prediction performance. As a result, it is discovered that the combination of two numerical features and two categorical features maintains a c-index of 0.714 (mean) and 0.018 (std) in the training set, with slightly reduced performance in the test set, yielding values of 0.711, 0.754, and 0.733 for c-index, c-index IPCW, and AUCD-ROC respectively. Significantly, the interpretability concordance index (ICI) improves to 0.476, indicating that this is the most balanced model produced by the SCI-XAI. The selected features for this model include “ejection_fraction”, “serum_creatinine”, “diabetes”, and “sex”.

**Table 7:** Survival performance results with training set (cross-validation approach) and test set (new unseen data) with feature selection.

Classifier	c-Index	#F	c-Index	c-Index IPWC	AUCD_ROC.	ICI
Cox proportional hazard	0.700 (0.048)	5(N), 4(C)	0.658	0.670	0.669	0.175
Cox proportional hazard IPCW	0.704 (0.051)	2(N), 5(C)	0.647	0.646	0.654	0.293
Random survival forest	0.706 (0.035)	4(N), 4(C)	0.675	0.723	0.684	0.235
Extra survival trees	0.696 (0.054)	4(N), 1(C)	0.657	0.704	0.666	0.406
Survival support vector machine	0.698 (0.057)	3(N), 5(C)	0.638	0.631	0.649	0.233
Gradient boosting models	0.714 (0.013)	2(N), 5(C)	0.724	0.762	0.748	0.298
Gradient boosting models*	0.714 (0.018)	2(N), 2(C)	0.711	0.754	0.733	0.476

C-index, concordance index; C-index IPCW, concordance index inverse probability of censoring weights; AUCD_ROC, area under the cumulative/dynamic ROC; ICI, interpretability concordance index.

*Model extracted by manual inspection.

### 4.5. Explainability analysis of the classification prediction model

As for the explainability assessment of the ensemble trees algorithms considered in this work, the HF survival prediction model built with the Random Forest classifier demonstrates the most balanced model in terms of explainability and accuracy. Therefore, in this subsection, the relevance of the following features “sex”, “ejection_fraction” and “serum_creatinine”, is analyzed to show their influence in the prediction task. As following, different *post hoc* explainability techniques are implemented on the selected prediction model.

**Figure 2:**
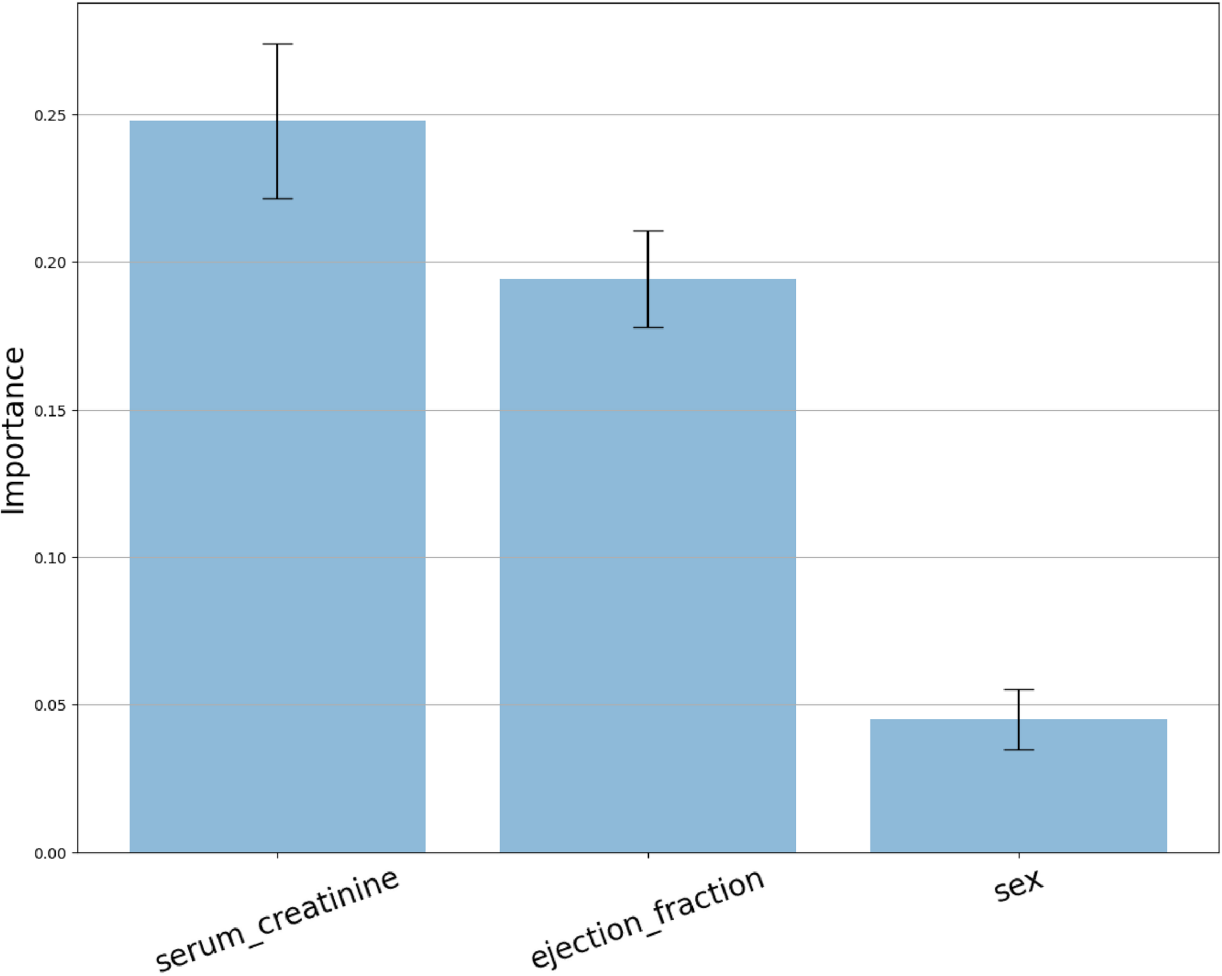
Feature permutation importance distribution.

Figure 2 demonstrates that permuting the values of the “serum_creatinine” feature results in the largest increase in prediction error, as compared to the other features. Therefore, the feature permutation technique identifies “serum_creatinine” (mean: 0.248, std: 0.026) as the most relevant feature, followed by “ejection_fraction” (mean: 0.194, std: 0.016), and “sex” (mean: 0.045, std: 0.010) in decreasing order of importance.

**Figure 3:**
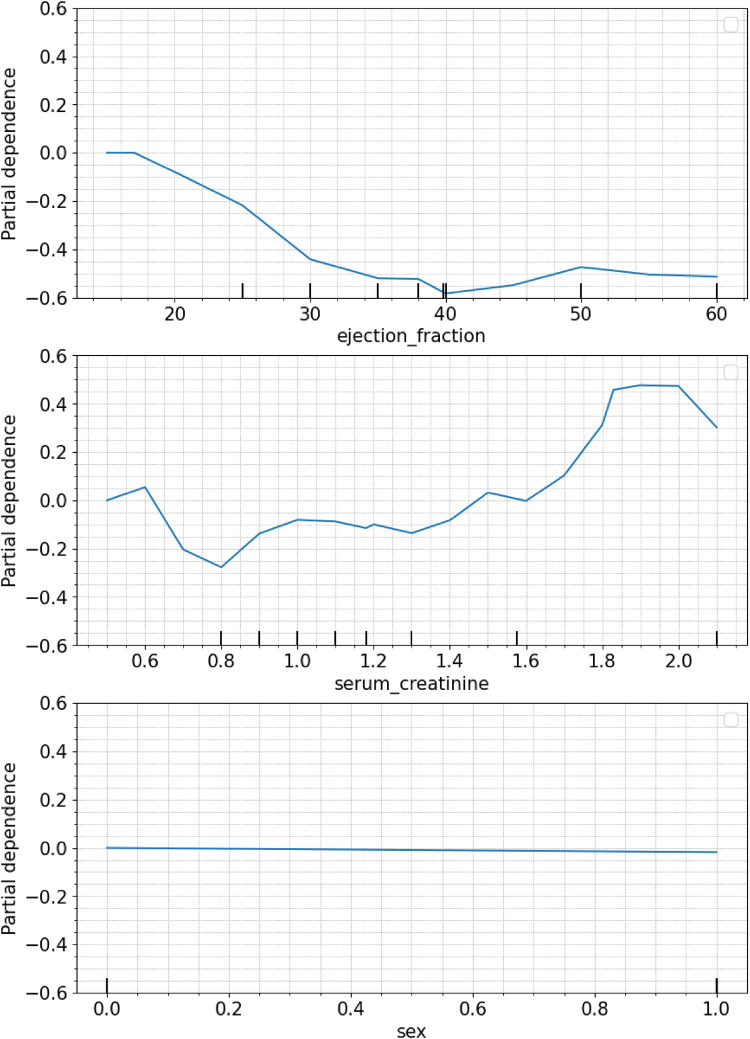
Individual PDP of the features selected: ejection fraction (top), serum creatinine (middle), sex (bottom).

The PDP *post hoc* visual explanation provides insights into the trend of marginal effect or the direction of influence between the target feature and the distribution values of the features selected in the model: “ejection_fraction” (Figure 3. top), “serum_creatinine” (Figure 3. middle), and “sex” (Figure 3. bottom). By exploring the PDP curve, experts can identify specific values at which the marginal effect curve changes, enabling them to establish certain thresholds, intervals or trigger values that affect the prediction probability. In addition, negatives values of the curve manifest an inverse influence (negative probability) on the target outcome and vice versa. Regarding “ejection_fraction”, its influence remains below 0 for every value of their distributions exhibits a moderate negative slope for values between 0% and 35%, which continuing monotonic between 35% and 39%, to drop down to −0.6 at 40%. Between 40% and 60% the marginal effect increases slightly up to 0.5. The feature “serum_creatinine” presents a varied distribution accross its values, where a threshold point at 1.45 mg/dl distinguishes between positive and negative contribution to the prediction of death. Values below 1.45 mg/dl result in a reduced marginal effect on the probability of death, having in the value 0.8 mg/dl the largest negative contribution of −0.3. Conversely, for values above the threshold, a quadratic increase can be observed, with a contribution of 0.5 at a value of 1.85 mg/dl. In the case of “sex”, the PDP plot does not show a significant effect neither for male or female subjects.

**Figure 4:**
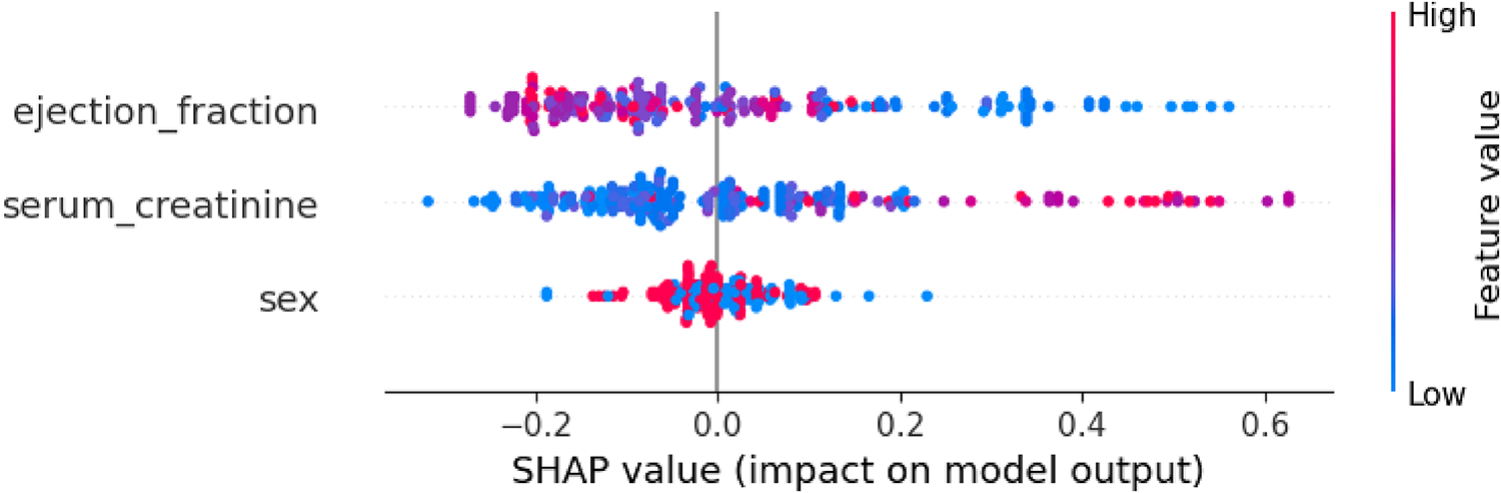
Global explanability of HF death prediction model by using SHAP.

By using the SHAP library (77, 85), the Shapley values technique can be applied to analyze global explainability for a specific classifier, in this case, Random forest. SHAP allows depicting the influence for the prediction of each of feature's values contained in the dataset. Figure 4 shows the importance of each feature represented by the width of the dots groups, as well as showing the positive or negative influence according to the features' values (red: high values, blue: low values). This overall plot provides insights into the influence of the model, aligning with the findings from the other XAI techniques. Specifically, it shows that feature “serum_creatinine” has the most significant contribution, as indicated by the width of its dots ranging from −0.35 to 0.65, where high values of the feature correspond with positive prediction of death while low values have small and even negative influence in the prediction. In the case of “ejection_fraction” is slightly less relevant than “serum_creatinine”, and low values are associated with positive contribution to death, while high values have a negative impact. On the other hand, the feature “sex” has the least contribution compared to “ejection_fraction” and “serum_creatinine” and there is no clear difference in the prediction contribution according to its values (male/female).

### 4.6. Explainability analysis of the survival analysis model

**Figure 5:**
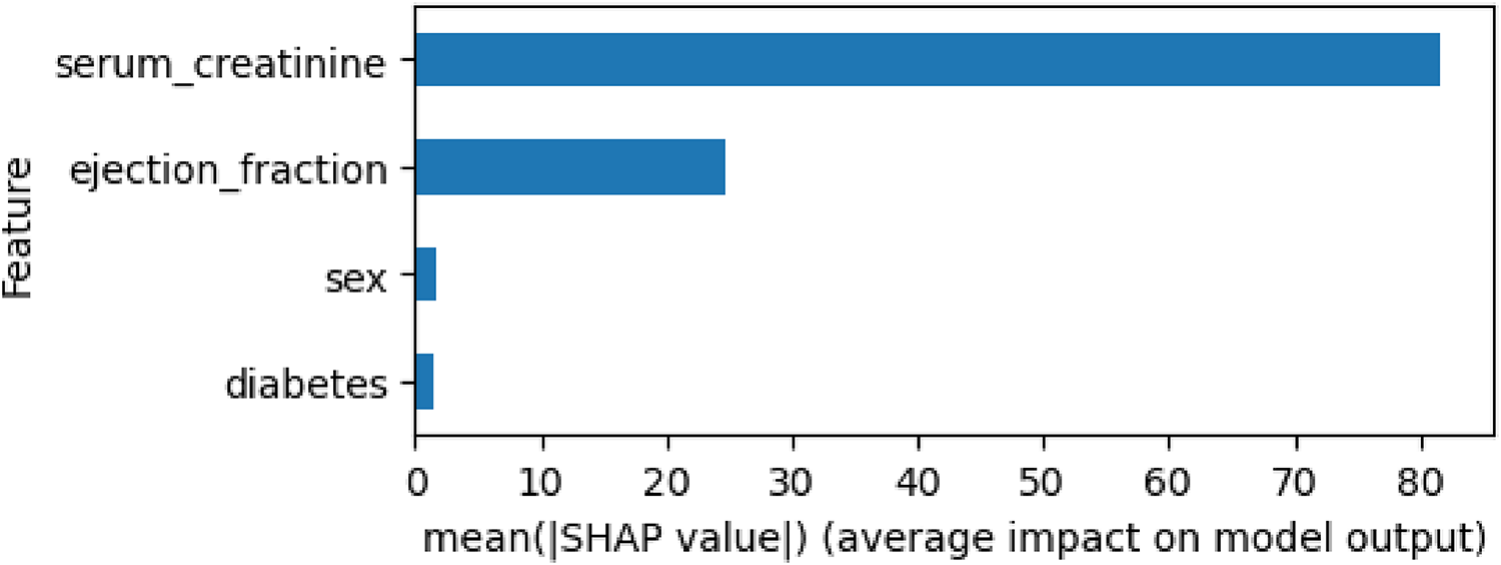
Global explanability of HF survival analysis by using SurvSHAP.

**Figure 6:**
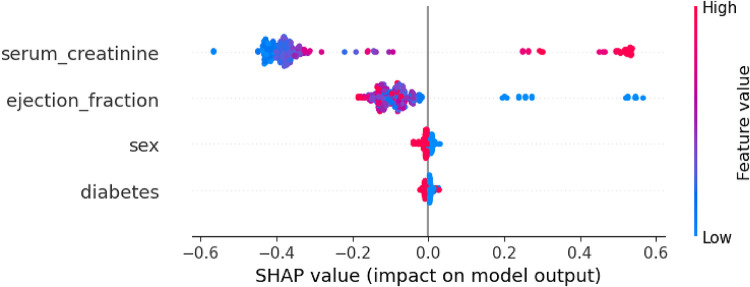
Global explanability of HF survival analysis according to the values of the features selected by using SurvSHAP.

Similarly to the prediction model as a classification problem, the explainability of the survival model can also be analyzed using SHAP. The SurvSHAP library enables the generation of plots illustrating the global explainability of the different features considered, expressed either in absolute values (Figure 5) or according to the features' values (Figure 6). In this analysis, “serum_creatinine” emerges as the most relevant feature for survival prediction, while “ejection_fraction” exhibits significantly less relevance, accounting for less than half of the importance. As for the categorical features “sex” and “diabetes,” their contribution to the survival prediction is deemed negligible. By considering the values of these features, Figure 6 displays high values of “serum_creatinine” positively contribute to the prediction of the event (death) in the survival analysis, while low values can be associated with a negative contribution to the event occurrence. Regarding “ejection_fraction”, high values are associated with slightly negative contribution to the death prediction, while low values positively influence the prediction of death. On the other hand, “sex” and “diabetes” does not present a substantial dispersion of their value dots that can be associated for any direction of the dead prediction.

## 5. Discussion

Due to the importance of CVD in the current global context of chronic diseases increase, the prediction of their outcomes like survival or disease onset by applying ML could has become a priority for doctors to achieve early identification of those factors related to the disease's effects. Moreover, XAI represents an advance to those prediction models by addressing clinicians' understandability requirements on the decision made by the models. XAI can also contribute to widening the prediction models' adoption in clinical practice since the professionals are enabled to make more reasonable and data-driven decisions. With more explainable clinical prediction models, doctors could focus on controlling those underlying features or indicators, and trying to reverse the worsening condition of patients who has suffered from HF in this case.

This paper aims to describe a prediction model for HF survival that facilitate the early detection of indicators leading to death events. The prediction model has been developed through two-fold approach. First, the problem is treated as a classification problem, where the target is to predict the occurrence of the death event without considering the time variable in the dataset. Second, the problem is addressed as a survival analysis, where the prediction aims to identify the event of death along with the influence of time on the occurrence of the event. The development of these models have consider not only on high prediction performance but also on analyzing the explainability of the results. This research contributes to enlarge the works dedicated to HF survival prediction by using ML through a novelty perspective, to the best of our knowledge, that tackle the model's explainability as a relevant part of the overall approach either if the problem is treated as a classification approach or a survival analysis. By employing *post hoc* explainability techniques, this work support “opening” the black-box paradigm of the ensemble trees classifiers employed in clinical prediction models.

The prediction model for HF survival has been developed using a data management optimization pipeline that was previously developed by the authors. This pipeline automates the data preprocessing, modeling, and evaluation phases, allowing for the automatic determination of various parameters such as the optimal ML algorithm and relevant features selected. This approach helps to find the optimal prediction model in terms of prediction performance and interpretability, while improving its efficiency and scalability. To ensure the model's robustness in predicting new unseen data, the pipeline performs a double evaluation of the model's performance by initially splitting the dataset for training and testing purposes.

The dataset employed for building the prediction model includes the target outcome feature “death_event”, which indicates whether the patient has died or not during the follow-up period after experiencing a heart attack. Another feature in the dataset is “time”, which represents the number of days of follow-up undergone by the patient until a death event occurs or the patient ends the follow-up period (censoring). These two variables allow for approaching the prediction problem as a typical survival analysis, where the target features are the event to predict (death in our case) and the time-to-event or censoring. Therefore, the SCI-XAI pipeline have been implemented by considering various machine learning algorithm dedicated to survival analysis, and a set of metrics that allow for measuring the prediction performance of the model, i.e., c-index, c-index ipcw and AUCD_ROC. The pipeline was trained through a 5 fold cross-validation approach, where the c-index was used as a metric to identify the best combination of feature selection and the survival analysis algorithm, including Cox proportional hazard, Cox proportional hazard IPCW, Random Survival Forest, Extra Survival Trees, Survival SVM, and Gradient Boosting Models. The results obtained indicate that the Gradient Boosting model perform the best in terms of prediction performance, with a mean c-index of 0.714 (std 0.013) during the cross-validation step. After evaluating these algorithms on unseen data (test set), the Gradient Boosting models continue to achieve the highest performance with a c-index of 0.724, c-index IPCW of 0.762, and AUCD_ROC of 0.748. To find a more balanced model in terms of prediction performance and interpretability, manual exploration of the top 10 combinations found by SCI-XAI was conducted. This search aimed to identify a model with high performance and fewer selected features, quantified by the Interpretability Concordance Index (ICI). As a result, another Gradient Boosting model that utilized only four features (“serum_creatinine”, “ejection_fraction”, “sex”, and “diabetes”) was identified. This model demonstrated similar performance during cross-validation and slightly lower performance on unseen data. However, it achieved the highest ICI value of 0.476 among all the algorithms, indicating superior balance between prediction performance and interpretability.

However, considering that the majority of related works have approached this dataset using a classification machine learning approach, we have also decided to apply the SCI-XAI pipeline to predict the occurrence of death using an optimal combination of ensemble tree methods. These methods include Random Forest, Extra Trees, AdaBoost, Gradient Boosting, and XGBoost, along with feature selection techniques. For model training and validation, we employed a 5-fold cross-validation approach. The best classification results belong to Random forest with an accuracy and balanced accuracy of 0.78 (std. 0.02) and 0.74 (std. 0.03). We must note that due to the imbalance in the target feature (203 for *y* = 0 and 96 for *y* = 1), the balanced accuracy is used to obtain the best model in the optimization SCI-XAI pipeline. Furthermore, classification performance generally decreases when dealing with instances in the test set. In the case of test set evaluation, Random Forest maintains the best results with 0.75 and 0.71 of accuracy and balanced accuracy, respectively. It is worth highlighting that the test set comprises 30% of the entire dataset, which could emulate a deployment environment where the model encounter new unseen data. However, the model's performance in an actual clinical environment might differ from the results due to the inherent complexities of medical records, which often contain a large number of features and more intricate patterns. When benchmarking these results with the related works identify, our prediction model does not achieve outperforming the models, which present a higher accuracy due to, in part, that authors include time as a predictor when training the models. Nevertheless, our contribution to these works is an extensive analysis of the explainability of the results by using *post hoc* XAI techniques, which, to the best of our knowledge, has not been carried out in the literature.

The results obtained demonstrate the effectiveness of the SCI-XAI pipeline in identifying relevant features when building the prediction models, whether using the survival analysis approach or the classification approach. The best models, which strike a balance between performance and interpretability, achieve a significant reduction in the number of original features (from eleven to four in the survival analysis and from eleven to three in the classification approach). In both approaches, the selected features are consistent. The features “serum creatinine,” “ejection_fraction,” and “sex” are selected in both cases, and “diabetes” is also selected for the survival analysis. The reduction in the number of features and its impact on model performance are quantified using the Interpretability Concordance Index (ICI) for the survival analysis and the Interpretability-Accuracy Index (IAI) for the classification approach. These indexes enable benchmarking of different machine learning techniques and facilitate the selection of the most balanced models for analyzing the explainability of their predictions' logic.

Regarding the explainability analysis of the prediction model developed using both approaches, the insights obtained regarding the relevance of the features are consistent. The feature “serum creatinine” emerges as the most influential feature for predicting death cases in both the classification and survival analysis. The feature “ejection_fraction” is identified as the second most important feature, while the impact of “sex” and “diabetes” on the prediction outcome is relatively small. The consistency in the influence of the features is also observed when exploring the values of the features. PDP and SHAP plots provide valuable insights into the direction of the influence on the prediction based on the feature values. For example, in both approaches, high values of “serum creatinine” are associated with a positive prediction of the death event, while lower values have a smaller or even negative influence on the prediction. PDP plots offer an opportunity to identify thresholds, intervals, or specific feature values where a certain feature may significantly increase or decrease the probability of the prediction. This implies that doctors can consider treatments or interventions to adjust patient features to safer values that decrease the probability of the predicted outcome, such as a death event. In this work, a threshold value of 1.45 mg/dl for “serum creatinine” has been identified, where the marginal effect of the feature changes its direction from negative to positive towards the predicted outcome, i.e., the event of death.

Therefore, the results described in this work demonstrate the added value of explainability to clinical prediction models. Additionally, by utilizing *post hoc* explainability techniques and feature selection, the baseline prediction model that deals with all features of the original dataset is improved not only in terms of prediction performance but also explainability. In addition, by offering a balance between these two aspects, the prediction model for HF survival could serve as a valuable tool for healthcare experts and increase its possibilities for being adopted in clinical routine.

## 6. Limitations of the study

Despite the interesting insights achieved in this work, which could enhance the prediction of survival after an HF event, some limitations might hinder the feasibility of generalizing the results to a broader population.

Firstly, the dataset was collected in an urban area of Pakistan, which may have substantial differences in terms of population features (poor quality of life, access to healthcare services, life expectancy) compared to rural areas of the country. Therefore, the application of the results to other populations within the country should be taken cautiously. In addition, Pakistan is a developing country where access to healthcare services is not comparable to Western societies. Consequently, the prediction model may yield different results if applied to another dataset collected from a developed country. This highlights another limitation of the study because although the SCI-XAI pipeline establishes an initial stratified split to create a train and test set enhancing the generalizability of the model with unseen data, the prediction model is trained and tested by using the same dataset. Therefore, it would be recommended to use another dataset collected in a different population location-wise to assess the generalizability of the model and reduce the inherent bias associated with employing the same dataset for training and testing purposes.

Additionally, the distribution of the target feature presents a substantial imbalance that may bias the model's performance toward predicting false positives for survival patients. Therefore, oversampling techniques like SMOTE could be used to balance the ratio of the target feature and mitigate this data collection bias. However, it is important to note that by doing so, the actual distribution of survival patients may be altered, leading to a prediction model that misclassifies patients when deployed in a real clinical setting. To address this concern, we recommend involving HF experts in the decision-making process regarding the oversampling approach. Their input can help ensure that the oversampling technique does not create a non-representative sample of the survival population after HF.

From the reviewed works, only a few have addressed the prediction of death events as survival analysis using the dataset employed in this study. While survival analysis is a well-known problem in statistics with various applications in healthcare and other fields, there is a limited literature and lack of tools that approach survival analysis from a machine learning perspective, using algorithms commonly employed in classification and regression problems. This scarcity of resources hampers the implementation of approaches for conducting comprehensive explainability analyses of prediction models' results. However, with the emergence of new tools and techniques for survival machine learning models, the findings obtained in this study could be further refined and improved, providing more insightful conclusions regarding the importance of the features in survival prediction.

Furthermore, the fact that the authors have a sole data science profile highlights the criticality of involving HF experts in this study. Their participation is essential for interpreting the results from a clinical standpoint, especially regarding XAI. This limitation also impacts the practicality of implementing the findings in a clinical setting, where the clinical validation of the XAI outcomes, including the determination of value thresholds from the PDP diagrams, becomes vital for the adoption of the prediction model. Therefore, future work will focus on engaging HF experts to strengthen the clinical validation of the obtained results. By incorporating their expertise, we aim to enhance the interpretation and applicability of the model's outcomes in real-world clinical scenarios.

## 7. Conclusions

This work presents the development and evaluation of explainable prediction models for HF survival considering a dual approach, first, addressing the survival prediction through a survival analysis and through a classical ML classification problem. With the aim of demonstrating the importance of considering explainability in early diagnosis clinical systems based on machine learning, the prediction models developed are improved by adopting a balanced compromise between the model's classification performance and its explainability, which could make it more suitable for its adoption in clinical practice.

Through an automated data management optimization pipeline, the best combination of the ML algorithm, i.e., ensemble trees algorithms for classification approach, and survival ML techniques for survival analysis, and the number of features selected for the model can be identified. Moreover, different evaluations based on prediction performance and explainability metrics to detect the best-balanced model in terms of prediction and explainability. Therefore, the explainable prediction model identified for the survival analysis approach is a Survival gradient boosting model over the following four features “serum_creatinine” (level of creatinine in the blood), “ejection_fraction” (percentage of blood leaving the heart at each contraction), “diabetes” (if the patient has diabetes), “sex” (gender of the patient). Furthermore, the classification problem approach determines the Random Forest with the following three features “serum_creatinine”, “ejection_fraction”, and “sex” as the optimal model.

The novelty presented by this work is the explainability approach adopted in the both prediction models for HF survival (classification and survival analysis), aiming to facilitate healthcare professionals' understanding and interpretation of the model's outcomes. By adopting this approach, clinicians can early identify changes in a patient's health using a smaller set of indicators and focus on treating those relevant features to potentially prevent adverse outcomes that put patient's survival at risk.

In future works, it could be beneficial to test the prediction model developed in a clinical setting to assess the robustness of the model in terms of accuracy with new patients' data. Additionally, gathering feedback from healthcare professionals regarding the explainability of the model's results would provide valuable insights for further improvement and refinement.

## Data Availability

Publicly available datasets were analyzed in this study. This data can be found here: https://archive.ics.uci.edu/ml/datasets/Heart+failure+clinical+records.

## Footnotes

^1^The source code of this work can be found in https://github.com/petmoreno/Heart_Failure_Predictor.
