# Supplementary figures and images for: Improvement of a prediction model for heart failure survival through explainable artificial intelligence
Source: Front Cardiovasc Med. 2023 Aug 1;10:1219586. doi: 10.3389/fcvm.2023.1219586 (PMC10434534; doi:10.3389/fcvm.2023.1219586)

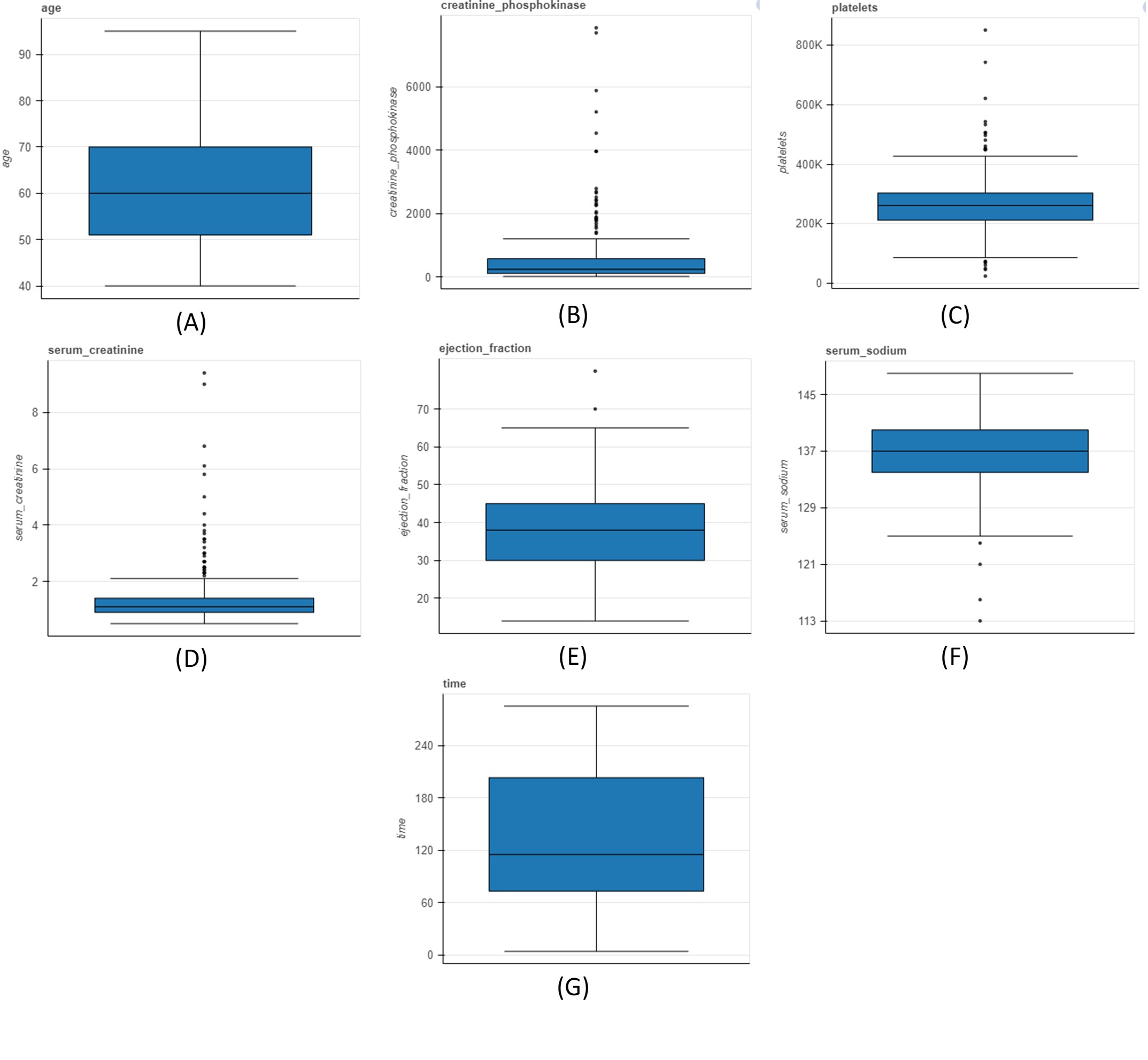

Supplement: Supplementary file 1 [file Image1.jpeg]

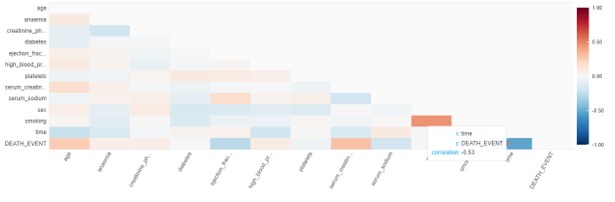

Supplement: Supplementary file 2 [file Image2.jpeg]
